# The burden of extrahepatic organ failures in European patients with cirrhosis

**DOI:** 10.1097/HC9.0000000000000832

**Published:** 2025-12-01

**Authors:** Wenyi Gu, Thierry Artzner, Lise Lotte Gluud, Salvatore Piano, William Bernal, Wim Laleman, Minneke J. Coenraad, Paolo Angeli, Jonel Trebicka, Richard Moreau

**Affiliations:** 1 Department of Internal Medicine B, Faculty of Medicine, Muenster University, Muenster, Germany; 2 Hôpitaux Universitaires de Strasbourg, Strasbourg, France; 3 Gastro Unit, Copenhagen University Hospital, Hvidovre, Denmark; 4 Department of Clinical Medicine, Faculty of Health Sciences, University of Copenhagen, Copenhagen, Denmark; 5 Unit of Internal Medicine and Hepatology, Department of Medicine, University and Hospital of Padova, Padova, Italy; 6 Institute of Liver Studies, King's College Hospital, University of London, London, England; 7 Department of Gastroenterology and Hepatology, Section of Liver and Biliopancreatic Disorders and Liver Transplantation, University Hospitals Leuven, KU Leuven, Leuven, Belgium; 8 Department of Gastroenterology and Hepatology, Leiden University Medical Center, Leiden, The Netherlands; 9 European Foundation for the Study of Chronic Liver Failure, Barcelona, Spain; 10 Centre de Recherche sur l’Inflammation, Institut National de la Santé et de la Recherche Médicale and Université Paris-Cité, Paris, France; 11 Service d’Hépatologie, Assistance Publique-Hôpitaux de Paris, Hôpital Beaujon, Clichy, France

**Keywords:** cirrhosis, disease burden, healthcare system, mortality, organ failure

## Abstract

**Background:**

The impact of acute-on-chronic liver failure (ACLF), a deadly form of decompensated cirrhosis characterized by the presence of organ failures, has not been well characterized, largely due to the lack of a code for ACLF in the International Classification of Diseases (ICD). We used ICD codes for extrahepatic organ failure to assess the burden of cirrhosis with extrahepatic organ failures (EHOFs) on European health care systems.

**Methods:**

The authors have searched national healthcare system databases from Germany for the period 2005–2020 and matched the data with that from France, Italy, and Denmark for admissions between 2017 and 2020, specifically for cases with an ICD diagnosis of cirrhosis combined with kidney, brain, respiratory, or circulatory failure.

**Results:**

During the 4-year period, 1,599,680 hospital admissions for cirrhosis, which included 329,093 (20.6%) admissions with at least 1 EHOF, were recorded across the 4 countries. The most frequent failing organs were kidneys (52.9%) and respiration (41.2%). The annual number of admissions for cirrhosis decreased over time (from 414,093 to 375,112), whereas the percentage of admissions with EHOF rose from 19.9% to 21.5%. Overall, the in-hospital mortality rate of patients with a diagnosis of EHOF was high (29.2%), markedly exceeding the mortality of those with a diagnosis of cirrhosis (7.9%). The proportion of estimated total healthcare claims of all hospital admissions of EHOF from cirrhosis was 44.9%.

**Conclusions:**

This study reveals that the burden of cirrhosis with EHOF was high in the 4 European countries, with a substantial impact on patient mortality. Crucially, these findings underscore the significant economic strain placed on healthcare systems by EHOF in cirrhosis patients. This should motivate all stakeholders to take action aiming at reducing this burden.

## Graphical abstract

**Figure:**
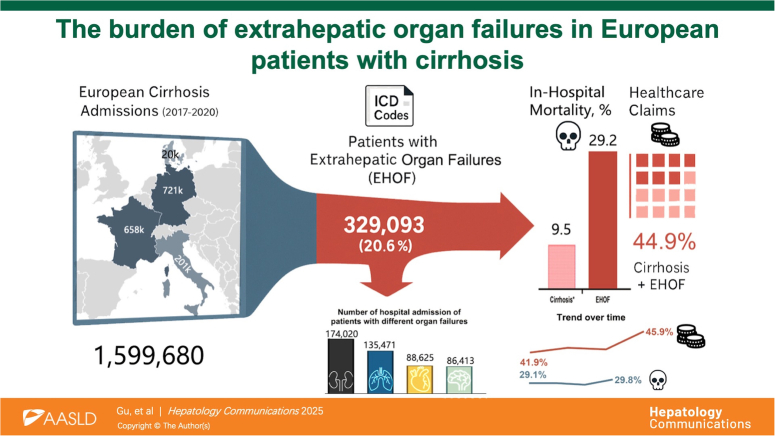

## INTRODUCTION

The term “acute-on-chronic liver failure” (ACLF) used in this article refers to the empirically proven concept, which has been substantiated with the use of results obtained in the CANONIC study.^1^ This study was a large European observational prospective study, which was specifically designed to determine diagnostic criteria of ACLF among a population of patients non-electively admitted for acutely decompensated cirrhosis (ADC). Because of the robustness of the methodology used in the CANONIC study, the subsequent ACLF definition developed from the results of this study is now globally accepted and adopted.^2^,^3^ The CANONIC study has shown that ACLF is the most severe form of ADC, characterized by the presence of at least 1 organ system failure (involving liver, kidney, brain, coagulation, circulation, respiration) and a high risk of death within 3 months.^1^ The 28-day case-fatality rate ranged from 19% for patients with a single kidney failure to 89% for patients with 4–6 organ failures.^1^ Currently, the management of ACLF is limited,^2^,^4^ either to identify and treat urgently the precipitants (eg, acute bacterial or fungal infection, acute liver damage caused by alcohol-related hepatitis) or provide organ support. The best curative treatment of ACLF is liver transplantation, even for patients with ≥2 organ failures.^4^ A recent meta-analysis of cohort studies using homogenous diagnostic criteria for ACLF reported that 4 in 10 patients admitted with decompensated cirrhosis had ACLF and that 6 in 10 patients with ACLF died by 90 days,^3^ findings that indicated a high burden of ACLF in Europe and worldwide.

Surprisingly, there are very few published data on the impact of ACLF on European healthcare systems. In addition, it is unclear how extraordinary challenges of the social and healthcare systems (eg, COVID-19 pandemic) impact outcomes in such a vulnerable population. This knowledge gap may at least in part be explained by the absence of code for ACLF in the *International Classification of Diseases*, even in the *9th* and *10th revisions* (ICD-9 and ICD-10). Nevertheless, ICD-9/10 codes are available for extrahepatic organ failures (EHOF) and provide a proxy for ACLF. For that reason, we decided to search national health system databases from Germany 2005–2020, France, Italy, and Denmark for diagnoses of EHOFs in patients with cirrhosis admitted between 2017 and 2020.

## METHODS

### Study design and data collection

We searched national healthcare system databases from Germany, France, Italy, and Denmark for hospital admissions to intensive care units (ICUs) or general wards, identifying cases with ICD codes, operation and procedure (OPS) (in Germany) and common classification of medical acts (CCAM) codes for cirrhosis and associated organ failures, specifically kidney, brain, respiratory, and circulatory failures (Table 1). We were able to analyze hospital admission data from 2005 to 2020 in Germany and align the data from 2017 to 2020 with that of France, Italy, and Denmark. This study utilized anonymized, aggregated, summary-level data, and no individual-level or identifiable private information was accessed or analyzed.

**TABLE 1:** ICD-10-GM and ICD-9-CM codes for cirrhosis and each organ failure are considered in the present study

Variable	ICD-10-GM^a^		ICD-9-CM^b^	
Cirrhosis	K74 or K70.3	Fibrosis and cirrhosis of the liver; alcoholic cirrhosis of the liver	571.5 or 571.2 or 571.6	Cirrhosis of the liver without mention of alcohol; alcoholic cirrhosis of the liver; biliary cirrhosis
Brain failure	Grade 3^c^: K72.73	Grade 3 hepatic encephalopathy: somnolence to stupor; response to verbal stimuli; confusion; disorientation to time and space	572.2	Hepatic coma
	Grade 4^c^: K72.74	Grade 4 hepatic encephalopathy: coma; no response to verbal stimuli or pain stimulation		
	R40^d^	Coma		
Kidney failure	K76.7 or N17.-, or 8-85a^e^	Hepatorenal syndrome, acute renal failure	572.4	Hepatorenal syndrome
		Acute kidney failure	584	Acute kidney failure
		Dialysis procedure due to a lack of function and failure of a kidney transplant	39.95 or 54.98	Hemodialysis; peritoneal dialysis
Circulation failure	R57 or	Cardiogenic shock, hypovolemic shock, septic shock, and other forms of shock	785.50, 785.51, 785.52, or 785.59	Shock, unspecified; cardiogenic shock; septic shock; other shock without mention of trauma
	8-931^e^	Monitoring of respiration, heart, and circulation with measurement of central venous pressure	89.62 or 89.64	Central venous pressure; pulmonary artery/wedge pressure
	EQLF001 or EQLF003^f^	Use of vasopressors		
Respiratory failure	J96.0 or	Acute respiratory failure, not elsewhere classified	518.81	Acute respiratory failure
	8-71^e^	Mechanical ventilation	96.71 or 96.72 or 96.05 or 96.7	Continuous invasive mechanical ventilation; Other intubation of the respiratory tract; other continuous invasive mechanical ventilation
	GLLD004, GLLD007 GLLD008, GLLD009 GLLD012, GLLD015^f^			

^a^ ICD-10-GM: International Classification of Diseases, 10th Revision, German Modification.

^b^ ICD-9-CM: International Classification of Diseases, 9th Revision, Clinical Modification.

^c^ ICD-10 code for hepatic encephalopathy was used only in Germany.

^d^ ICD-10 code for coma was used in France and Denmark.

^e^ OPS code for dialysis, measurement of central venous pressure, or mechanical ventilation in Germany.

^f^ CCAM code for vasopressors or mechanical ventilation was used in France.

Abbreviations: CCAM, common classification of medical acts; OPS, operation and procedure.

In our study, we used ICD-10 codes in Germany, France, and Denmark, while ICD-9-CM was employed in Italy. The slower transition from ICD-9 to ICD-10 in Italy is partly due to regional variations in healthcare system administration and infrastructure, which is managed by Italy's decentralized National Health Service (SSN). For cirrhosis, ICD-10-GM codes K74 and K70.3 were utilized, whereas in Italy, we relied on ICD-9-CM codes 571.5, 571.2, and 571.6. For hepatic encephalopathy (brain failure), the following ICD-10-GM codes were used: K72.73 (Grade 3 hepatic encephalopathy) and K72.74 (Grade 4 hepatic encephalopathy) in Germany. A code for coma with R40 was used as a substitute for brain failure in France. Similarly, hepatic coma (R40.2) was used in Denmark. Italy’s ICD-9-CM system used the code 572.2 for coma. In the assessment of kidney failure, we used ICD-10-GM codes K76.7, N17 and OPS 8-85a, while ICD-9-CM/OPS used the codes 572.4, 584.9, 39.95, and 54.98. For circulatory failure, the ICD-10-GM included R57 for various forms of shock, alongside OPS 8-931. In ICD-9-CM, shock-related conditions were recorded using 785.50, 785.51, 785.52, or 785.59, with central venous and pulmonary artery pressure monitoring coded as 89.62 and 89.64, respectively. Lastly, respiratory failure was defined using J96.0 and 8-71. While in Italy, the equivalent codes were 518.81 and 96.71, 96.72, 96.05, and 96.7. Details for the organ failure definition and data source were described in Supplementary Methods.

Due to the limitations of ICD coding, liver failure and coagulation failure (intrahepatic organ failure) were not included in our analysis. Liver failure, in particular, is challenging to define using ICD codes, as it often captures cases of acute liver failure or jaundice (total bilirubin >5 mg/dL), which do not meet the criteria for cirrhosis-related liver failure (total bilirubin >12 mg/dL). Similarly, coagulation failure, defined as INR >2.5, lacks a distinct code in the Diagnosis-Related Group (DRG) system, which instead includes conditions such as vitamin K deficiency or acquired coagulation factor deficiency.

We also investigated the key demographic patterns, including age and sex, as well as etiologies of cirrhosis (alcohol-related or non-alcohol-related) for each case. Complications associated with cirrhosis and EHOF were collected from Germany (Supplemental Table S1, http://links.lww.com/HC9/C180). Finally, we examined the in-hospital mortality rates across all countries, the length of hospital stays from Germany, France, and Denmark, and the healthcare claims data for each hospital stay from Germany, France, and Italy. These data were unavailable from Denmark. These demographic and disease burden data were compared with hospital admissions of patients diagnosed with cirrhosis, where such data were available.

### Statistical analysis

Descriptive statistics, including the number and proportions of hospital admissions by diagnoses, sex, etiologies, and in-hospital mortality, were reported. Age distributions were described using means and SDs. In addition, medians and IQRs were reported for these parameters to summarize central tendencies and variability. For variables where cross-country data could not be directly aggregated, such as median healthcare claims (in Euros) and length of hospital stay (in days), a weighted median was employed to account for differences in data availability and sample sizes across countries. Temporal trends were assessed by stratifying the data by year while combining the countries. Year-specific analyses were performed to detect any changes in admissions or outcomes over time. The year 2020 was analyzed separately due to the profound impact of the COVID-19 pandemic on healthcare systems and hospital utilization during this period.

Cross-country comparisons were conducted to examine differences in demographic characteristics, clinical outcomes, and healthcare resource utilization among the 4 countries.

Analyses and figures were performed using GraphPad Prism, with results presented as numbers (percentages), means ± SD for normally distributed variables and medians (IQR) or weighted medians for non-normally distributed variables.

## RESULTS

### Prevalence of EHOF

**FIGURE 1:**
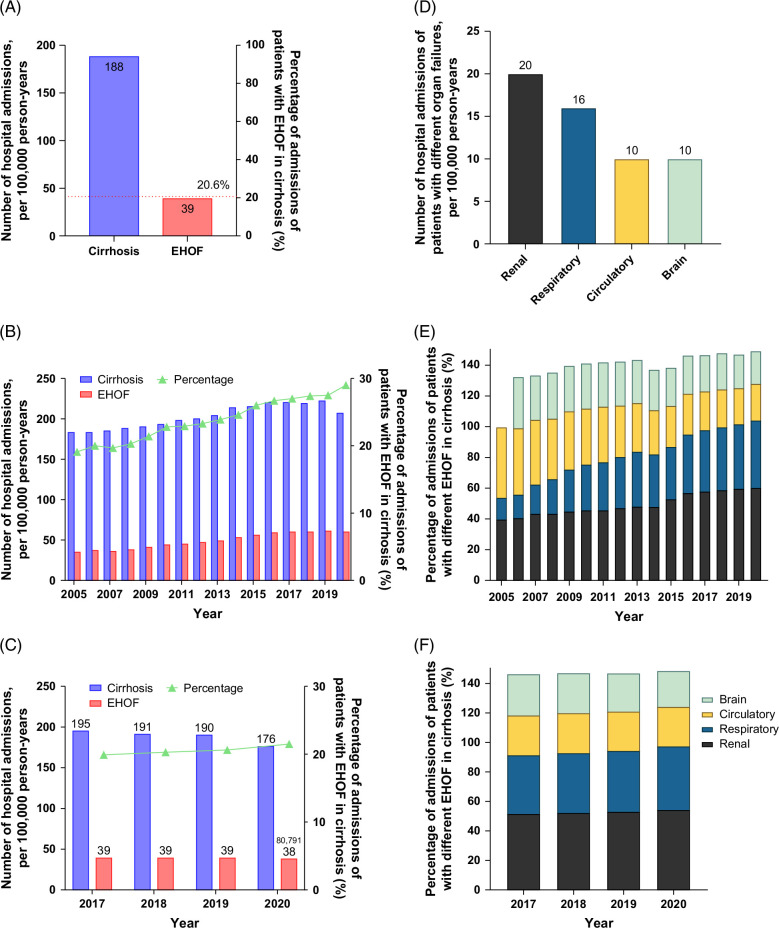
Disease burden of cirrhosis and extrahepatic organ failures. (A) The number of hospital admissions of patients diagnosed with cirrhosis or diagnosed with extrahepatic organ failure in cirrhosis, expressed as per 100,000 person-years, and the percentage of patients with extrahepatic organ failure in cirrhosis from Germany, France, Italy, and Denmark. (B) Trends in the number of hospital admissions, expressed as per 100,000 person-years, and percentage of admissions of patients diagnosed with extrahepatic organ failure in cirrhosis from Germany over the years 2005–2020. (C) Trends in the number of hospital admissions, expressed as per 100,000 person-years, and percentage of admissions of patients diagnosed with extrahepatic organ failure in cirrhosis from Germany, France, Italy, and Denmark over the years 2017–2020. (D) The number of admissions for different organ failures (renal, respiratory, circulatory, and brain) in cirrhosis patients from Germany, France, Italy, and Denmark, expressed as per 100,000 person-years. (E) Time trend of the percentage of admissions with specific extrahepatic organ failures in cirrhosis from Germany over the years 2005–2020. *Note:* Hepatic encephalopathy was accurately coded in Germany from the year 2006 onward. (F) Time trend of the percentage of admissions with specific extrahepatic organ failures in cirrhosis from Germany, France, Italy, and Denmark over the years 2017–2020. Abbreviation: EHOF, extrahepatic organ failure.

Between 2017 and 2020, a total of 1,599,680 admissions for cirrhosis were recorded across 4 European countries. The overall incidence of cirrhosis-related hospitalizations during this period was 187.9 per 100,000 person-years (Table 2). During that period, a total of 329,093 (20.6%) admissions for cirrhosis with EHOF were identified, corresponding to an incidence rate of 38.7 per 100,000 person-years (Figure 1A).

**TABLE 2:** General characteristics of all hospital admissions combining the data from Germany, France, Italy, and Denmark from the year 2017 to 2020

Characteristics	All hospital admissions of patients
Population, n	851,327,718
Cirrhosis	
Cirrhosis, n	1,599,680
Cirrhosis, n/100,000 population	187.9
Age^a^, mean	63.8
Male^a^, %	68.6
EHOF	
EHOF, n	329,093
EHOF, %	20.6
EHOF, n/100,000 inhabitants	38.7
Age, mean	64.9
Male, %	68.3
Alcohol related	
Alcohol-related cirrhosis, n (%)	865,345 (54.1)
Age^a^, mean	62.5
Male^a^, %	76.0
Alcohol-related EHOF, n (%)	189,637 (21.9)
Age^a^, mean	62.4
Male^a^, %	74.5
Organ failures, n (%)	
Renal failure	174,020 (52.9)
Respiratory failure	135,471 (41.2)
Circulatory failure	88,625 (26.9)
Brain failure	86,413 (26.3)
Number of EHOF^b^, n (%)	
One-organ failure	218,656 (66.7)
Two-organ failures	69,374 (21.2)
Three-organ failures	32,732 (10.0)
Four-organ failures	6996 (2.1)
Length of stay, median	
Cirrhosis^c^	7
EHOF	12
Total healthcare claim from all admissions	
Cirrhosis (Euro)^c^	4,767,046,400
EHOF (Euro)^d^	2,138,117,221
Proportion of EHOF from cirrhosis (%)	44.9
In-hospital mortality (%)	
Cirrhosis	7.9
Alcohol-related cirrhosis	8.5
EHOF	29.2
Alcohol-related EHOF^a^	29.9

^a^ Mean age and male percentage of patients diagnosed with cirrhosis were calculated using the mean age and male number from Germany, France, and Italy.

^b^ The number and percentage of different number of organ failures in EHOF and in-hospital mortality of alcohol-related EHOF was calculated using the numbers from Germany, France, and Italy.

^c^ Median length of hospital stay and total healthcare claim of patients coded with cirrhosis were estimated using the median and IQR of the data from Germany and France.

^d^ Total healthcare claim for hospital stays of patients coded with EHOF was estimated using the median and IQR of the data from Germany, France, and Italy.

Abbreviation: EHOF, extrahepatic organ failure.

The proportion of EHOF among cirrhosis admissions in Germany rose significantly from 19.1% in 2005 to 29.1% in 2020 (Figure 1B), findings suggesting that the progressive increase in admissions for EOHF observed between 2017 and 2020 in the 4 European countries might reflect a “natural” trend rather than an “accident” related to this latter period (Supplemental Figure S1, http://links.lww.com/HC9/C180).

The total annual admissions for cirrhosis decreased by 2.4 percentage points, from 194.8 per 100,000 person-years in 2017 to 189.7 per 100,000 person-years in 2019, followed by a more substantial decline of 9.4 percentage points, to 176.2 per 100,000 person-years in 2020 (Figure 1C). These changes may be attributable to the effects of the COVID-19 pandemic. In contrast, the yearly number and percentage of admissions for EHOF in cirrhosis increased from 38.8 per 100,000 person-years (19.9%) in 2017 to 39.2 per 100,000 person-years (20.6%) in 2019 and escalated further to 21.5% in 2020 (Figure 1C).

### Demographic characteristics and variations

The mean age of patients admitted with cirrhosis was 63.8 years, while it was 64.9 years among those diagnosed with additional EHOF (Table 2). The average age of patients admitted with EHOF was stable (around 65 years old) throughout the study period (Table 3).

**TABLE 3:** Characteristics of all hospital admissions in each year of 2017, 2018, 2019, and 2020, combining the data from Germany, France, Italy, and Denmark

	Years			
Characteristics	2017	2018	2019	2020
Population, n	212,532,839	212,822,176	213,049,599	212,923,104
Cirrhosis				
Cirrhosis admission, n	414,117	406,319	404,132	375,112
Cirrhosis, n/100,000 person-years	194.8	190.9	189.7	176.2
Age^a^, mean (SD)	63.6	63.6	64.1	64.3
Male^a^, %	68.3	68.5	68.6	69.0
EHOF				
EHOF, n	82,535	82,324	83,443	80,791
EHOF, %	19.9	20.3	20.6	21.5
EHOF, n/100,000 person-years	38.8	38.7	39.2	37.9
Age, mean	64.9	64.9	65.7	64.9
Male, %	68.3	68.2	68.6	68.3
Alcohol related				
Alcohol-related cirrhosis, n (%)	223,503 (54.0)	219,085 (53.9)	216,900 (53.7)	205,857 (54.9)
Alcohol-related EHOF, n (%)	47,534 (21.6)	47,487 (22.0)	47,846 (22.4)	46,770 (23.0)
Organ failures, n (%)				
Renal failure, n	42,663	43,051	44,282	44,024
%	51.7	52.3	53.1	54.5
Brain failure, n	22,847	22,440	21,600	19,526
%	27.7	27.3	25.9	24.2
Respiratory failure, n	32,833	33,408	34,443	34,787
%	39.8	40.6	41.3	43.1
Circulatory failure, n	22,448	22,285	22,261	21,631
%	27.2	27.1	26.7	26.8
Number of EHOF^b^				
One-organ failure, n	55,465	54,722	55,468	53,001
%	67.2	66.5	66.5	65.6
Two-organ failures, n	16,853	17,349	17,725	17,447
%	20.4	21.1	21.2	21.6
Three-organ failures, n	8054	8170	8213	8295
%	9.8	9.9	9.8	10.3
Four-organ failures, n	1795	1755	1699	1747
%	2.2	2.1	2.0	2.2
Length of stay, days, median				
Cirrhosis^c^	7	7	7	7
EHOF	12	12	12	11
Total healthcare claim of all admissions, Euro				
Cirrhosis^c^	1,234,068,660	1,251,056,201	1,219,266,244	976,791,648
EHOF^d^	516,834,170	534,859,028	559,902,530	398,622,794
Proportion of EHOF from cirrhosis (%)	41.9	42.8	45.9	40.8
In-hospital mortality (%)				
Cirrhosis	8.4	8.5	8.4	8.9
Alcohol-related cirrhosis	9.1	9.2	9.0	9.5
EHOF	29.1	29.1	28.8	29.8
Alcohol-related EHOF^a^	29.5	29.9	29.2	30.4

^a^ Mean age and male percentage of patients coded with cirrhosis were calculated using the mean age and male number of each year from Germany, France, and Italy.

^b^ The number and percentage of different numbers of organ failure in EHOF and in-hospital mortality of alcohol-related EHOF were calculated using the numbers from each year from Germany, France, and Italy.

^c^ Median length and total healthcare claims of hospital stay of patients coded with cirrhosis were estimated using the median and IQR of the data from Germany and France.

^d^ Total healthcare claim for hospital stays of patients coded with EHOF was estimated using the median and IQR of the data from Germany, France, and Italy.

Abbreviation: EHOF, extrahepatic organ failure.

The majority (68.6%) of patients admitted with cirrhosis were males. Similarly, among patients admitted with EHOF, 68.3% were male, showing no significant deviation from the overall cohort of patients with cirrhosis. The proportion of male patients remained relatively stable over time, ranging from 68.3% to 68.6%.

### Types of organ failures

Regarding each type of EHOF, kidney failure was the prominent EHOF, representing 52.9% (174,020) of all organ failures across the 4 years and among all 4 countries. Respiratory failure followed, representing 41.2% (135,471) of cases, and circulatory failure was observed in 26.9% (88,625) of admissions. Cerebral failure had the lowest prevalence, contributing to 26.3% (86,413) of all EHOF cases (Figure 1D).

In Germany, the proportion of hospital admissions due to respiratory and renal failure among EHOF patients significantly increased from 14.1% and 39.8%, respectively, in 2005 to 43.9% and 60.3%, respectively, in 2020. In contrast, the proportions of cerebral and circulatory failure declined by nearly 20% (Figure 1E). Similarly, across the 4 Central European countries, the incidence of renal failure gradually increased over the study period, rising from 51.5% in 2017 to 53.1% in 2019, and further to 54.5% in 2020 (Figure 1F and Supplemental Figure S2, http://links.lww.com/HC9/C180). Respiratory failure also demonstrated a 3 percentage points increase, from 39.8% in 2017 to 41.3% in 2019, and 43.1% in 2020. In contrast, the prevalence of circulatory failure remained relatively stable, while cerebral failure decreased by 3.5% during the same period (Figure 1F).

The majority of hospital admissions for patients with cirrhosis and EHOF involved a single-organ failure, which accounted for 66.7% (218,656) of all cases (Table 2). Two-organ failures were identified in 21.2% (69,374) of admissions, while only 12.1% (19,864) of cases involved 3 or more organ failures. Despite year-to-year variations in the types of organ failures, the distribution of patients with single, 2, 3, or more organ failures remained relatively stable throughout the study period (Table 3).

### Alcohol-related cirrhosis and EHOF with different etiologies

Alcohol-related liver disease (ArLD) was the leading etiology of cirrhosis, accounting for 54.1% (865,345) of admissions attributed to alcohol consumption. Among these cases, 21.9% (189,637) were complicated by the presence of EHOF. The overall prevalence of alcohol-related cirrhosis remained stable at ~54% throughout the study period from 2017 to 2020. However, the proportion of admissions with EHOF in alcohol-related cirrhosis increased from 21.6% in 2017 to 22.4% in 2019, with a further rise to 23.0% in 2020, a trend that may be partially attributable to the effects of the COVID-19 pandemic (Table 2).

This proportion varied markedly between northern and southern Europe. Denmark reported the highest prevalence of alcohol-related cirrhosis, although it decreased from 67% in 2017 to 64.7% in 2020. In contrast, Italy showed a rising trend, from 34.5% in 2017 to 40.0% in 2020. Germany reported an increase of 1.8 percentage points, France by 1.1 percentage points, and Italy by 1 percentage point (Supplemental Table S2, http://links.lww.com/HC9/C180).

In addition, we analyzed data on HCV and HBV as etiologies of cirrhosis with EHOF. Our findings indicate a marked decrease in admissions for both HCV (from 6.5% to 2.3%) and HBV (from 2.2% to 1.1%) as underlying causes (Supplemental Table S3, http://links.lww.com/HC9/C180).

### In-hospital mortality and complications

**FIGURE 2:**
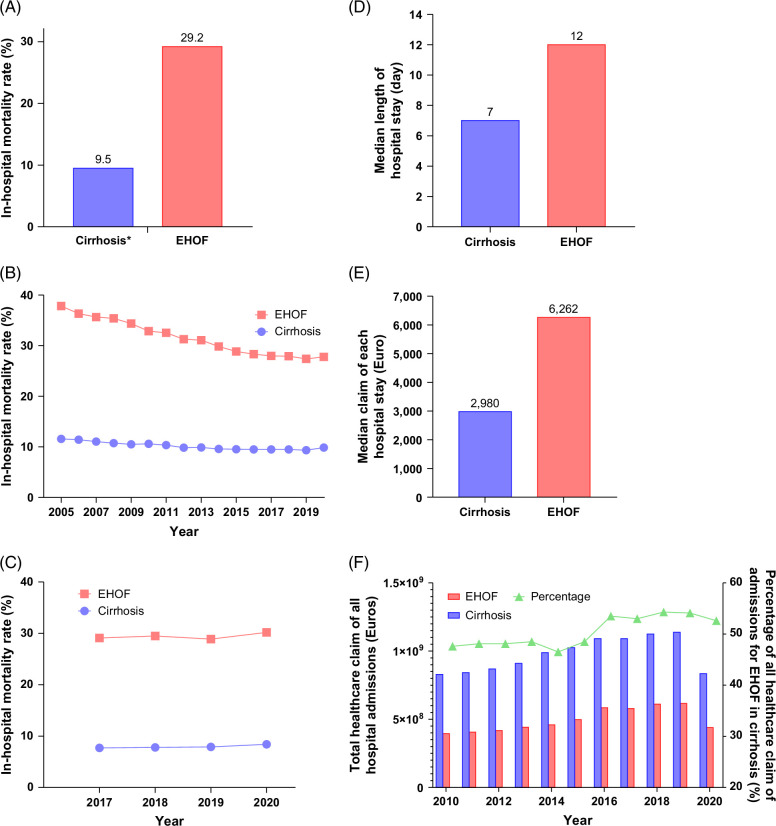
In-hospital mortality, hospital stay, and healthcare claims for cirrhosis and EHOF. (A) In-hospital mortality rates for Germany, France, Italy, and Denmark. (B) Trends in in-hospital mortality rates in Germany from 2005 to 2020. (C) Trends in in-hospital mortality rates in Germany, France, Italy, and Denmark from 2017 to 2020. (D) Median length of hospital stay for cirrhosis patients and those with EHOF across all hospital admissions. (E) Median healthcare claims in euros for hospital stay for cirrhosis patients and those with EHOF. The median length of stay and healthcare claims were estimated using the median and IQR of data from Germany and France from 2017 to 2020. (F) Total healthcare claims in euros for all hospital admissions of patients diagnosed with cirrhosis or EHOF, as well as the proportion of total healthcare claims in euros for admissions of patients diagnosed with EHOF relative to those diagnosed with cirrhosis, from 2010 to 2020. Abbreviation: EHOF, extrahepatic organ failure.

The overall in-hospital mortality rate for patients admitted with cirrhosis was notably high at 7.9%. Among patients with cirrhosis, those with EHOF had a markedly elevated in-hospital mortality rate of 29.2%, more than 3 times higher than with cirrhosis (Figure 2A). A decreasing trend in mortality was observed in both EHOF and cirrhosis patients, with a notable reduction of 10% in mortality among EHOF patients, from 37.8% in 2005 to 27.8% in 2020 (Figure 2B). Across the 4 countries, the mortality rate remained relatively stable throughout the study period, fluctuating between 28.8% and 29.1%, before experiencing a slight increase to 29.8% in 2020 (Figure 2C). Notably, patients with alcohol-related cirrhosis had a marginally higher in-hospital mortality rate of 8.5% than the mortality rate of cirrhosis in general. Nevertheless, those developed alcohol-related EHOF demonstrated a mortality rate of 29.9%, exceeding the mortality rates of alcohol-related cirrhosis (Table 2).

Among all complications of cirrhosis and EHOF, ascites [increasing from 10,915 (37.8%) to 20,260 (50.4%) cases] was the most frequent complication in Germany, showing a substantial increase from 2005 to 2020, along with bacterial infections and hepatic encephalopathy, followed by hepatorenal syndrome. In contrast, varices and variceal bleeding were the least frequent complications (Supplemental Figure S3A, http://links.lww.com/HC9/C180). Using 2005 as the reference year, hospital admissions for EHOF patients due to spontaneous bacterial peritonitis (SBP) increased the most, reaching 3.95 times the 2005 level in 2020. This was followed by admissions for other bacterial infections and ascites. Notably, hospital admissions for varices and variceal bleeding dropped significantly in 2020, decreasing to 23% and 16% of their levels in 2005, respectively (Supplemental Figure S3B, http://links.lww.com/HC9/C180).

### Length of hospital stay and associated financial implications

The disease burden of cirrhosis and EHOF, particularly when there were multiple, was considerable in terms of length of stay and healthcare claims. From the DRG data of the 2 countries, the median hospital stay of patients with EHOF was 12 days, compared with a median of 7 days for the overall general admissions of patients with cirrhosis (Figure 2D) and Supplementary Figure S4A http://links.lww.com/HC9/C180.

The financial implications were similarly substantial. The total healthcare claim for hospital stays for all patients with EHOF accounted for almost half of the healthcare claims of those admitted with liver cirrhosis (44.9%) (Table 2 and Figure 2E). The proportion remained stable with a slight increase over the 3 years before COVID-19 (2017–2019), ranging from 41.8% to 45.9% (Table 3). In Germany, it has the highest proportion of healthcare claims of EHOF from cirrhosis admissions (60.4%), which is double the proportion from France (36.9%) (Supplemental Table S2 and Supplementary Figure S4B, http://links.lww.com/HC9/C180). We also assessed the proportion of healthcare claims for hospital admissions of patients with EHOF relative to all cirrhosis admissions each year. This proportion steadily increased from 47.6% in 2010 to 52.6% in 2020 in Germany. Of note, these healthcare claims were estimated using standard local reimbursement fees, which likely underestimate the true cost burden of EHOF in cirrhosis.

## DISCUSSION

This is the first study that attempts to describe the epidemiology of patients hospitalized with cirrhosis and EHOF over several European countries. Between 2017 and 2020, there were 329,093 admissions for cirrhosis with EHOF among the 4 countries included in this study. This represented 20.6% of the total admissions of patients with cirrhosis during this period. The most common EHOF was kidney failure, followed by respiratory, circulatory, and brain failure. In-hospital mortality rate was higher among patients with EHOF compared with the general population of patients with cirrhosis (29.2% vs. 7.9%, respectively). The median length of hospital stay and the median claim of each hospital stay were also higher among patients with EHOF compared with the general population of patients with cirrhosis.

Taken together, these results highlight 2 aspects of the burden of EHOF associated with cirrhosis. First, EHOF constitutes a burden for the healthcare system in terms of admissions, duration of stay, and costs. This epidemiological finding resonates with daily clinical practice. Namely, it is typically more demanding to treat patients with cirrhosis and EHOF than those without EHOF. From a public health perspective, awareness of this burden is pivotal to securing the appropriate allocation of resources to it. This also calls for trying to identify potential predictors of EHOF development among patients with cirrhosis in order to organize the management and, if possible, prevent the development of ACLF. The second aspect is that EHOF among patients with cirrhosis is associated with a considerable risk of in-hospital mortality. This is both consistent with studies focusing on ACLF,^3^,^5^,^6^ and it also echoes with clinical practice and pathophysiological findings. Decompensated cirrhosis associated with EHOF often takes the form of a systemic condition for which the individual treatment of each separate organ failure may fail to cure patients, especially those with multiple extrahepatic organ failures. A case in point is the decreased response to terlipressin associated with the increase in baseline ACLF grade.^7^ Liver transplantation can be a radical treatment in some cases of ACLF, but given the organ shortage and the vast number of admissions for cirrhosis and EHOF, it is very far from constituting a routinely available solution to the burden imposed by ACLF in Europe. In that respect, it seems crucial to try to unravel the pathophysiology of ACLF (in particular, the role of inflammation) that could potentially lead to developing new treatments with wider indications than those of liver transplantation.^8^,^9^,^10^,^11^

Using these registries was, in our opinion, the best way to grasp the burden of ACLF in Europe. That there are such obstacles to apprehending this issue is one of the key messages of this study. Indeed, ACLF has been identified as a potentially deadly condition that should call for dedicated medical resources. Matching adequate resources to medical demand requires being able to apprehend the size of the medical challenge that we face. This is also crucial for monitoring and benchmarking the quality of medical interventions across centers and countries. While this is possible in several other medical conditions for which dedicated clinical management networks already exist (eg, cancer, strokes, among others). We believe that collecting specific data on patients with ACLF and promoting dedicated clinical care networks should go hand in hand, as they both are necessary means to improve access to optimal care and reduce healthcare disparities. Such disparities have already been shown to exist in the specific context of liver transplantation for patients with severe ACLF^8^,^9^ and are likely to exist in other aspects of the treatment of ACLF (in particular, access to ICUs). The challenge raised by the burden of ACLF calls for raising awareness of the specificities of this deadly condition, both in terms of clinical management and in terms of scientific inquiry. It also calls for having additional and more granular epidemiological data on key issues related to ACLF, for example, precise data on infection, inflammatory markers, or types of respiratory failure.

A key finding was the vast difference in EHOF rates, particularly between Germany (27%) and Denmark (6.9%). We attribute this to fundamental healthcare system disparities. Germany’s DRG-based financing strongly promotes thorough coding of conditions like EHOF for reimbursement, a practice less prevalent under Denmark’s global budget system. In addition, Germany’s substantially higher hospital and ICU bed capacity may lower the threshold for documenting organ dysfunction, leading to higher observed EHOF rates compared with Denmark, where only the most severe cases might be captured. Another important epidemiological aspect beyond the scope of this study is the complex relationship between socioeconomic status and ACLF. Of note, our data showed that the healthcare claim of ACLF admissions accounted for almost half of those admissions due to cirrhosis. Two principal etiologies of chronic liver disease (CLD) and ACLF are closely related to socioeconomic status.^12^ First, there is a close association between low socioeconomic status and the incidence of ArLD.^13^,^14^,^15^,^16^ Second, metabolic dysfunction–associated steatotic liver disease (MASLD) is increasingly important as a cause of CLD and ACLF and its prevalence relates closely to the comorbidities, adverse health behaviors, poor diet and food insecurity that are more common in socially disadvantaged groups.^3^,^6^,^17^,^18^,^19^ Socioeconomic disadvantage in people with CLD is likely to impact access to and use of acute care and liver transplantation.^12^,^20^ Though specific data on its relation to ACLF is currently lacking, lower socioeconomic status (SES) is associated with adverse outcomes in other critical illnesses. People of lower SES are more likely to be admitted to critical care with sepsis, trauma, and the consequences of substance misuse,^21^,^22^ and when admitted, often have illness of greater severity and higher mortality.^23^,^24^,^25^ Of note, recent reports of liver transplantation of patients with ACLF have shown a close relation between severity of illness and measures of SES, with the most severe disease seen in those with the lowest SES.^26^

The key limitations of our analysis are related to encoding issues. First and foremost, the registries that were used are primarily intended and designed for insurance and reimbursement purposes, not for epidemiological studies. The second limitation is that there are clearly different ways and strategies of encoding between hospitals and countries. This entails that the differences that are observed between countries may, to a potentially large extent, be the product of encoding differences rather than actual clinical and epidemiological differences. Another limitation is the exclusion of liver and coagulation failures from our case-finding algorithm. However, we contend that this focused approach strengthens the internal validity, as the use of non-specific ICD codes would have introduced significant misclassification bias from cases of acute liver failure and non-hepatic coagulopathies, thereby compromising the stability of our findings. Notwithstanding these limitations, our study shed light on the fact that ICD codes for ACLF are an unmet need. Another limitation of our study was that it did not assess a subjective and qualitative aspect that almost certainly shapes the burden of ACLF stigma in the population of patients with cirrhosis. Stigma is the socially constructed phenomenon whereby one group is devalued by another on the basis of a recognized or perceived difference, and people with liver disease often belong to highly stigmatized groups.^6^ Stigma can exist on different levels—not only through the negative attitudes and discriminatory behaviors of the public and healthcare staff who perpetuate assumptions that CLD from ARLD, MASLD, or chronic viral infection is self-induced and less worthy of care and support, but also from self-stigma where people with CLD internalize these negative public attitudes with guilt, shame, and a low sense of self-worth.^6^,^27^,^28^ Stigma may result both in delayed presentation to care and in lower quality of care received, and evidence is emerging that this may be important in CLD and ACLF.^28^,^29^ Worryingly, this may impact decision-making in relation to admission to critical care.

This study reports epidemiological data that provides a first glimpse into the burden of EHOF in European patients with cirrhosis. It also highlights the efforts that lie ahead of us to have a better picture of this burden in order to effectively alleviate it.

## Supplementary Material
