# Supplementary material for: The burden of extrahepatic organ failures in European patients with cirrhosis
Source: Hepatol Commun. 2025 Dec 1;9(12):e0832. doi: 10.1097/HC9.0000000000000832 (PMC12668586; doi:10.1097/HC9.0000000000000832)
Supplement: Supplementary file 1 [file hc9-9-e0832-s001.docx]

Supplementary Appendix

Table Contents

|  | Contents | Page |
| --- | --- | --- |
| Supplementary Methods | Definition of each organ failure, data source and statistical analysis | 2 |
| Supplementary Figure 1 | Trends in regular hospital beds per 1,000 population, and ICU bed per 100,000 population capacity in Germany, France, Italy, and Denmark from 2017 to 2020, number or proportion of hospital admissions of patients diagnosed with EHOF of each country from cirrhosis or per 100,000 inhabitants from 2017 to 2020. | 4 |
| Supplementary Figure 2 | Proportion of each organ failure in EHOF of each country from 2017 to 2020 | 5 |
| Supplementary Figure 3 | Number and fold changes of complications of patients admitted with EHOF from Germany each year | 6 |
| Supplementary Figure 4 | Median length of hospital stay in days and median cost of each hospital stay of patients diagnosed with EHOF compared to those diagnosed with cirrhosis from Germany and France during 2017 to 2020. | 7 |
| Supplementary Table 1 | ICD-10-GM and definition of each complication associated with cirrhosis or EHOF from Germany | 8 |
| Supplementary Table 2 | Characteristics of all hospital admissions of patients diagnosed with cirrhosis or cirrhosis with EHOF from each country of Germany, France, Italy and Denmark, and from each year of 2017, 2018, 2019 and 2020. | 9 |
| Supplementary Table 3 | Number of hospital admissions of hepatitis C or hepatitis B patients diagnosed with cirrhosis and extrahepatic organ failure, and related proportion in all etiologies of extrahepatic organ failures | 10 |

**Supplementary Methods**

**Definition of each organ failure**

Renal failure was defined as hepatorenal syndrome, acute renal failure or kidney dialysis, and failure of a kidney transplant. Brain failure was defined as hepatic encephalopathy grade 3 or 4 from Germany, any grade of hepatic encephalopathy from Italy, and coma was used in France and Denmark. Circulation failure was defined using codes for the monitoring of respiration, heart and circulation with measurement of central venous pressure or pulmonary artery/wedge pressure, as well as the diagnosis code for cardiogenic shock, hypovolemic shock, septic shock and other forms of shock (or use of vasopressors [Denmark]). The definition for respiratory failure was diagnosis with acute respiratory failure or had procedure of mechanical ventilation. Since a patient could have only one main diagnosis but at most 89 secondary diagnoses for each admission. Both main diagnosis and secondary diagnosis were included.

In-hospital mortality was recoded as one reason from hospital discharge. The average length of stay was recorded as number of days in hospital until discharge or in-hospital mortality. The cost of each hospitalization was also included into the analysis.

**Data Source**

The data from Germany was acquired from RDC of the Federal Statistical Office and Statistical Offices of the Federal States of Germany, using controlled remote data processing using SAS scripts (SAS, version 3.8; SAS Institute Inc., Cary, NC). ICD-10-German Modification (GM) and German OPS from diagnosis-related groups (DRG) were used. For each observation in the data set, data are recorded during each hospitalization episode until discharge or in-hospital death.

The data from France was based on International Classification of Disease, 10th Revision, French Modification (ICD-10-FR), and CCAM Common Classification of Medical Acts.

The data from Denmark was extracted from the Danish Medical Classification System - Sundhedsvæsenets Klassifikations System (SKS), based on World Health Organization’s International Classification of Disease, currently ICD-10 version with national extensions, and classifications of treatments.

The data from Italy was used the code of International Classification of Diseases, 9th Revision, Clinical Modification (ICD-9-CM).

**Statistical analysis**

We report number and proportions of admissions with different diagnoses, sex, etiologies and number of in-hospital deaths of patients admitted to hospital. The age was reported by mean and standard deviation. We further report the median and interquartile range of age, length of hospital stay and cost of each stay.

**Supplementary Figure 1.** **A.** Trends in regular hospital beds per 1,000 population (dashed lines), and ICU bed per 100,000 population (solid lines) capacity in Germany, France, Italy, and Denmark from 2017 to 2020. Source: World Bank and OECD. **B.** Number of hospital admissions of patients diagnosed with EHOF of each country from 2017 to 2020; **C.** Proportion of hospital admissions of patients diagnosed with EHOF from cirrhosis of each country from 2017 to 2020; **D.** Number of hospital admissions of patients diagnosed with EHOF per 100,000 inhabitants of each country from 2017 to 2020. Abbreviation: EHOF, extra-hepatic organ failure.

**A B**


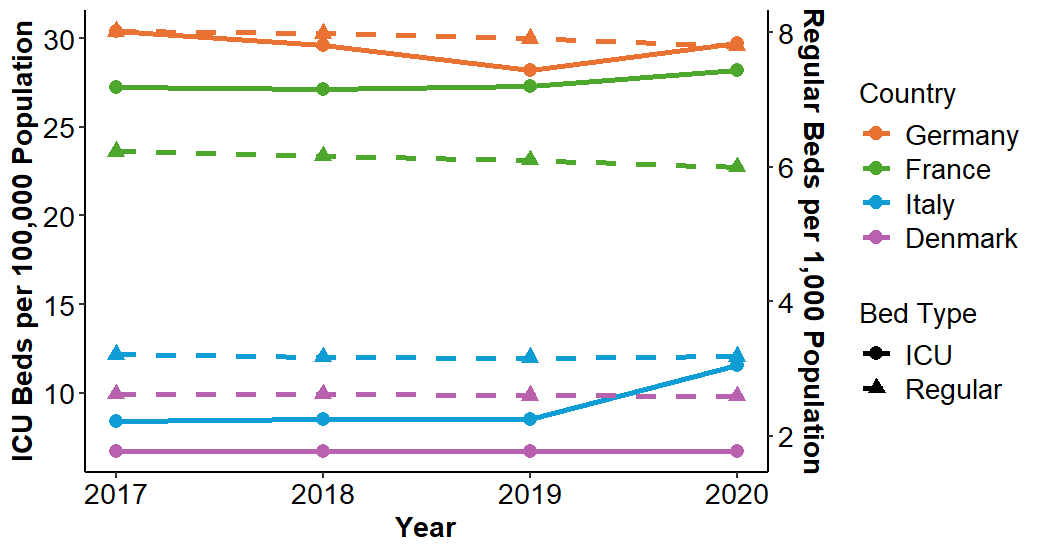

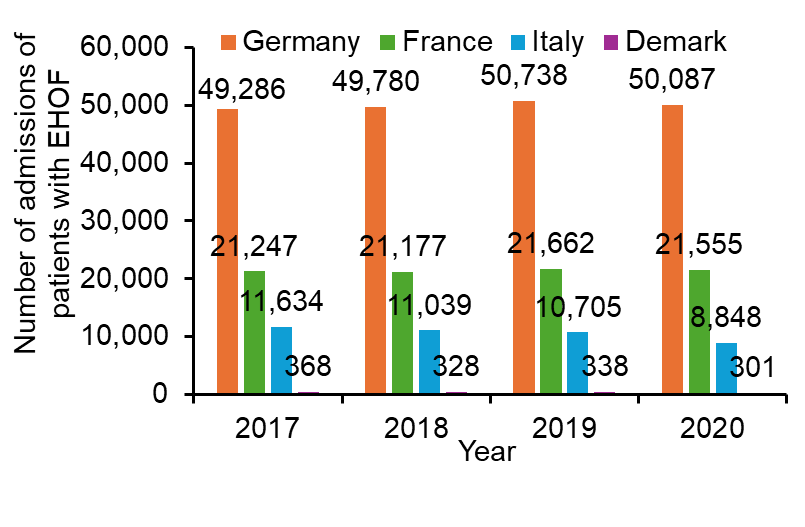


**C D**

**
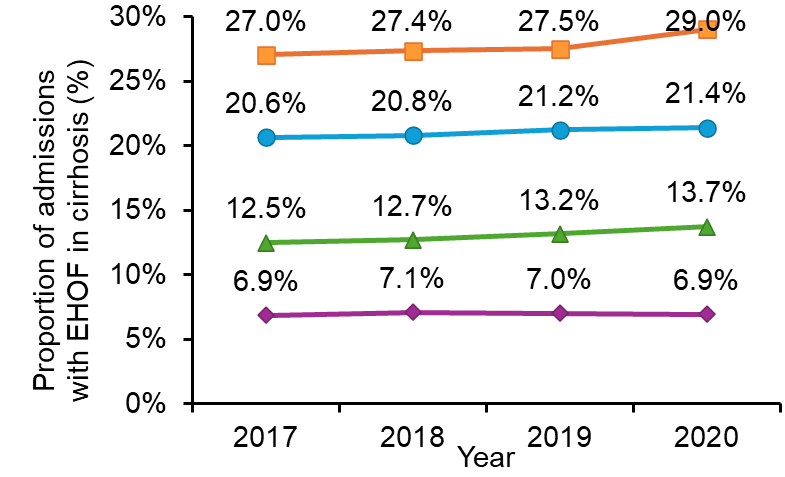
**
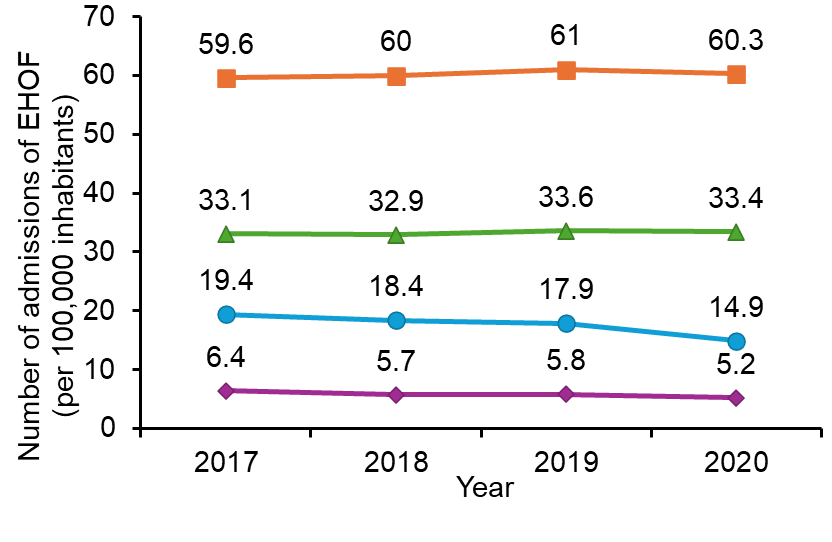


**Supplementary Figure 2**. Proportion of each organ failure in EHOF of each country from 2017 to 2020. Note: the diagnosis of EHOF was based on the ICD-code of cirrhosis and any of the four organ failures, including renal failure, brain failure, respiratory failure and circulatory failure. Abbreviation: EHOF, extra-hepatic organ failure.


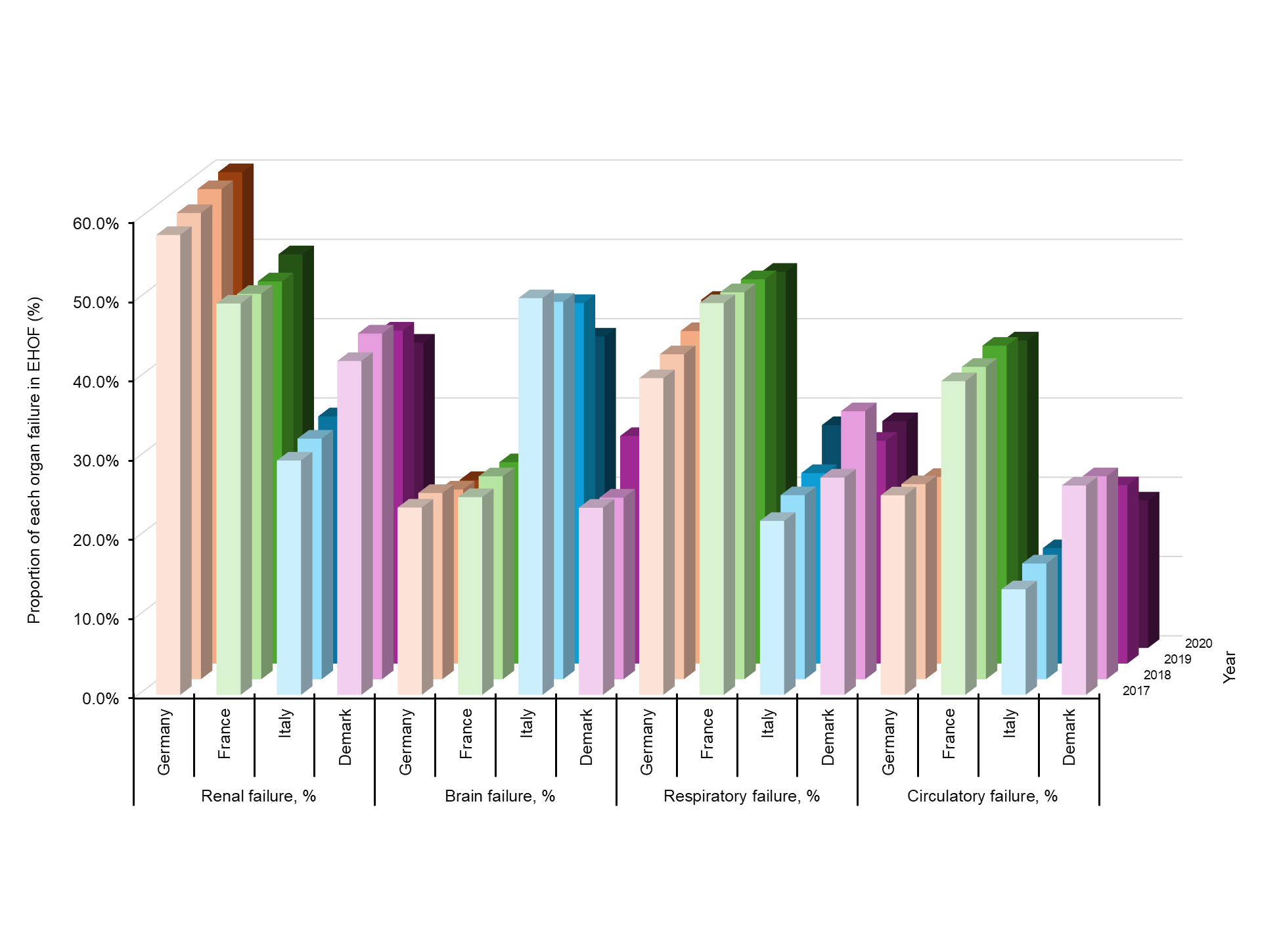


**Supplementary Figure 3.** **Number (Panel A) and fold changes (Panel B) of complications of patients admitted with EHOF from Germany each year.**

**A**

**B**

**Supplementary Figure 4. A.** Median length of hospital stay in days of patients diagnosed with EHOF compared to those diagnosed with cirrhosis from Germany and France during 2017 to 2020. **B.** Median cost of each hospital stay in Euro of patients diagnosed with EHOF compared to those diagnosed with cirrhosis from Germany and France during 2017 to 2020. Note: the diagnosis of EHOF was based on the ICD-code of cirrhosis and any of the four organ failures, including renal failure, brain failure, respiratory failure and circulatory failure. Abbreviation: EHOF, acute-on-chronic liver failure.

**A
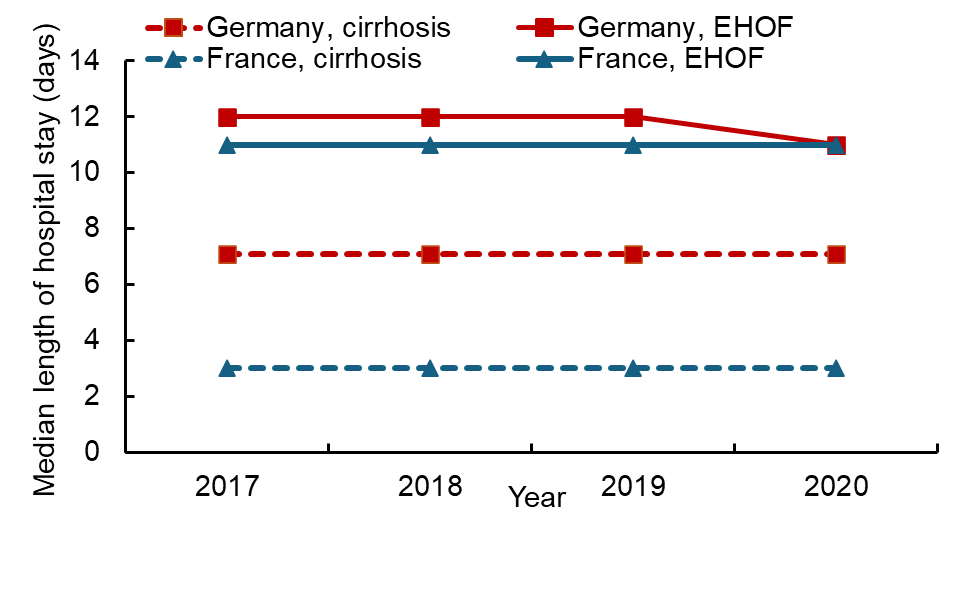
**

**B**


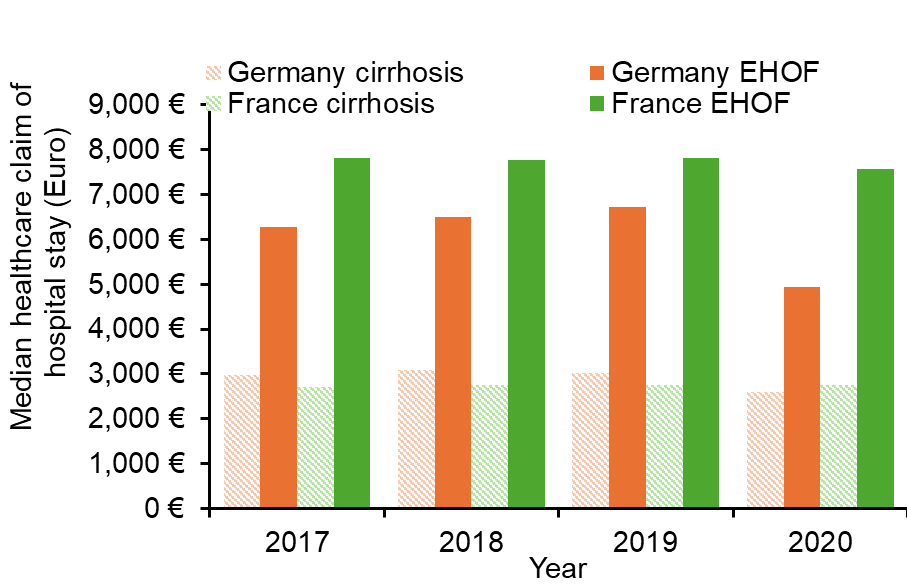


**Supplementary Table 1.** ICD-10-GM and definition of each complication associated with cirrhosis or EHOF from Germany.

| **Variable** | **ICD - 10** | **Definitions** |
| --- | --- | --- |
| **Liver cirrhosis** | K74, K70.3 | Fibrosis and cirrhosis of the liver and alcoholic cirrhosis of the liver |
| **Complications of liver cirrhosis** | |  |
| Ascites | R18 | Ascites  Includes: Fluid accumulation in the abdominal cavity |
| Hepatic encephalopathy | K72.7 | Hepatic encephalopathy |
| Infections | A00 to A09, A20 to A28, A30 to A89, A92 to A99, B95 to B98, N39, J12, J13, J14, J15, J16, J17, J18, L00, L01, L02, L03, L04, L05, L06, L07, L08, M72, K65, K83 | Infectious intestinal diseases; certain bacterial zoonoses; other bacterial diseases; infections that are mainly transmitted through sexual intercourse; other spirochete diseases; other diseases caused by chlamydia; rickettsioses; viral infections of the central nervous system; arthropod-borne viral diseases and viral hemorrhagic fevers; bacteria, viruses and other infectious agents as the cause of diseases that are classified in other chapters; pneumonia; urinary tract infection; skin and subcutaneous infections; fibromatoses and peritonitis and cholangitis. |
| Spontaneous bacterial peritonitis | K65.00 | Spontaneous bacterial peritonitis (acute) |
| Hepatorenal syndrome | K76.7 | Hepatorenal syndrome  Excludes: After labor and delivery |
| Esophageal varice | I85.9 | Esophageal varices without bleeding |
| Esophageal variceal bleeding | I85.0 | Esophageal varices with bleeding  Excludes: Esophageal varices with indication of bleeding in liver diseases and esophageal varices with indication of bleeding in schistosomiasis |
| Portal vein thrombosis | I81 | Portal vein thrombosis  Includes: Portal vein closure  Excludes: Portal vein phlebitis |

**Supplementary Table 2**. Characteristics of all hospital admissions of patients diagnosed with cirrhosis or cirrhosis with EHOF from each country of Germany, France, Italy and Denmark, and from each year of 2017, 2018, 2019 and 2020.

|  | Germany | | | | France | | | | Italy | | | | Denmark | | | |
| --- | --- | --- | --- | --- | --- | --- | --- | --- | --- | --- | --- | --- | --- | --- | --- | --- |
|  | 2017 | 2018 | 2019 | 2020 | 2017 | 2018 | 2019 | 2020 | 2017 | 2018 | 2019 | 2020 | 2017 | 2018 | 2019 | 2020 |
| Population, n | 82657002 | 82905782 | 83132799 | 83122889 | 64144086 | 64277808 | 64399759 | 64480053 | 60004032 | 59877425 | 59727932 | 59500579 | 5727719 | 5761161 | 5789109 | 5819583 |
| **Cirrhosis** | | | | | | | | | | | | | | | | |
| Cirrhosis admission, n | 182268 | 181856 | 184355 | 172478 | 170128 | 166747 | 164539 | 156941 | 56361 | 53087 | 50411 | 41340 | 5360 | 4629 | 4827 | 4353 |
| Cirrhosis, n/100,000 population | 220.5 | 219.4 | 221.8 | 207.5 | 265.2 | 259.4 | 255.5 | 243.4 | 93.9 | 88.7 | 84.4 | 69.5 | 93.6 | 80.3 | 83.4 | 74.8 |
| Age, mean (SD) | 64 (13) | 64 (13) | 65 (13) | 65 (13) | 62 (13) | 62 (13) | 62 (13) | 63 (13) | 67 (13) | 67 (16) | 68 (16) | 66 (12) |  |  |  |  |
| Male, % | 64.9 | 64.6 | 64.9 | 64.8 | 72.3 | 72.5 | 72.9 | 73.6 | 67 | 69 | 68 | 69 |  |  |  |  |
| **EHOF** | | | | | | | | | | | | | | | | |
| EHOF, n | 49286 | 49780 | 50738 | 50087 | 21247 | 21177 | 21662 | 21555 | 11634 | 11039 | 10705 | 8848 | 368 | 328 | 338 | 301 |
| EHOF, % | 27.0 | 27.4 | 27.5 | 29.0 | 12.5 | 12.7 | 13.2 | 13.7 | 20.6 | 20.8 | 21.2 | 21.4 | 6.9 | 7.1 | 7.0 | 6.9 |
| EHOF, n/100,000 person-year | 59.6 | 60.0 | 61.0 | 60.3 | 33.1 | 32.9 | 33.6 | 33.4 | 19.4 | 18.4 | 17.9 | 14.9 | 6.4 | 5.7 | 5.8 | 5.2 |
| Age, mean (SD) | 65(12) | 65(13) | 66 (13) | 65(13) | 63 (12) | 63 (12) | 64 (12) | 64 (12) | 68 (13) | 68 (16) | 68 (16) | 67 (12) | 63 (10) | 63 (10) | 63 (10) | 64 (10) |
| Male, % | 66.7 | 66.2 | 66.6 | 66.0 | 72.7 | 72.7 | 73.5 | 73.4 | 67 | 69 | 68 | 69 | 63.0 | 59.5 | 66.9 | 66.7 |
| **Alcohol related** | | | | | | | | | | | | | | | | |
| Alcohol related cirrhosis, n (%) | 96491 (52.9) | 95125 (52.3) | 94968 (51.5) | 89654 (52.0) | 103944 (61.1) | 101304 (60.8) | 99777 (60.6) | 96871 (61.7) | 19476 (34.5) | 19552 (37) | 19051 (38) | 16517 (40) | 3592 (67.0) | 3104 (67.1) | 3104 (64.3) | 2815 (64.7) |
| Age, mean (SD) | 62 (11) | 62 (11) | 62 (11) | 62 (11) | 62 (11) | 63 (11) | 63 (11) | 63 (11) | 63 (12) | 64 (19) | 63 (11) | 63 (11) |  |  |  |  |
| Male gender, % | 72.9 | 72.7 | 72.8 | 72.3 | 77.4 | 77.4 | 78.3 | 78.9 | 83 | 81 | 81 | 81 |  |  |  |  |
| Alcohol related EHOF, n | 28830 | 28782 | 28763 | 28462 | 14445 | 14274 | 14806 | 14540 | 4259 | 4431 | 4277 | 3768 |  |  |  |  |
| Alcohol related EHOF, % | 29.9 | 30.3 | 30.3 | 31.7 | 13.9 | 14.1 | 14.8 | 15.0 | 22 | 23 | 22.5 | 23 |  |  |  |  |
| Age, mean (SD) | 62 (11) | 62 (11) | 63 (11) | 62 (11) | 63 (11) | 63 (11) | 63 (11) | 63 (11) | 63 (12) | 64 (19) | 64 (11) | 62 (11) |  |  |  |  |
| Male gender, % | 72.4 | 72.1 | 72.3 | 71.7 | 77.0 | 76.6 | 77.5 | 78.1 | 83 | 81 | 81 | 81 |  |  |  |  |
| **Organ failures, n (%)** | | | | | | | | | | | | | | | | |
| Renal failure, n | 28585 | 29267 | 30360 | 30202 | 10485 | 10292 | 10442 | 10702 | 3438 | 3349 | 3338 | 3004 | 155 | 143 | 142 | 116 |
| % | 58.0 | 58.8 | 59.8 | 60.3 | 49.3 | 48.6 | 48.2 | 49.6 | 29.6 | 30.3 | 31.2 | 34.0 | 42.1 | 43.6 | 42.0 | 38.5 |
| Brain failure, n | 11642 | 11693 | 11135 | 10608 | 5298 | 5419 | 5501 | 5353 | 5820 | 5253 | 4867 | 3475 | 87 | 75 | 97 | 90 |
| % | 23.6 | 23.5 | 21.9 | 21.2 | 24.9 | 25.6 | 25.4 | 24.8 | 50.0 | 47.6 | 45.5 | 39.3 | 23.6 | 22.9 | 28.7 | 29.9 |
| Respiratory failure, n | 19683 | 20400 | 21277 | 21989 | 10497 | 10335 | 10499 | 10225 | 2552 | 2562 | 2572 | 2487 | 101 | 111 | 95 | 86 |
| % | 39.9 | 41.0 | 41.9 | 43.9 | 49.4 | 48.8 | 48.5 | 47.4 | 21.9 | 23.2 | 24.0 | 28.1 | 27.4 | 33.8 | 28.1 | 28.6 |
| Circulatory failure, n | 12398 | 12248 | 11944 | 11919 | 8405 | 8342 | 8683 | 8360 | 1548 | 1611 | 1558 | 1296 | 97 | 84 | 76 | 56 |
| % | 25.2 | 24.6 | 23.5 | 23.8 | 39.6 | 39.4 | 40.1 | 38.8 | 13.3 | 14.6 | 14.6 | 14.6 | 26.4 | 25.6 | 22.5 | 18.6 |
| **Number of extra hepatic organ failures** | | | | | | | | | | | | | | | | |
| One organ failure, n | 32618 | 32530 | 33370 | 32378 | 12765 | 12709 | 12874 | 13038 | 10082 | 9483 | 9224 | 7585 |  |  |  |  |
| % | 66.18 | 65.35 | 65.77 | 64.64 | 60.1 | 60.0 | 59.4 | 60.5 | 86.7 | 85.9 | 86.2 | 85.7 |  |  |  |  |
| Two organ failures, n | 11015 | 11400 | 11487 | 11584 | 4452 | 4565 | 4899 | 4747 | 1386 | 1384 | 1339 | 1116 |  |  |  |  |
| % | 22.35 | 22.9 | 22.64 | 23.13 | 21.0 | 21.6 | 22.6 | 22.0 | 11.9 | 12.5 | 12.5 | 12.6 |  |  |  |  |
| Three organ failures, n | 4790 | 4943 | 4975 | 5180 | 3104 | 3063 | 3103 | 2972 | 160 | 164 | 135 | 143 |  |  |  |  |
| % | 9.72 | 9.93 | 9.81 | 10.34 | 14.6 | 14.5 | 14.3 | 13.8 | 1.4 | 1.5 | 1.3 | 1.6 |  |  |  |  |
| Four organ failures, n | 863 | 907 | 906 | 945 | 926 | 840 | 786 | 798 | 6 | 8 | 7 | 4 |  |  |  |  |
| % | 1.75 | 1.82 | 1.79 | 1.89 | 4.4 | 4.0 | 3.6 | 3.7 | 0.1 | 0.1 | 0.1 | 0.0 |  |  |  |  |
| **Length of stay, median (IQR)** | | | | | | | | | | | | | | | | |
| Cirrhosis | 7  (3-13) | 7  (3-13) | 7  (3-13) | 7  (3-12) | 3  (0-9) | 3  (0-10) | 3  (0-10) | 3  (0-9) |  |  |  |  |  |  |  |  |
| EHOF | 12  (7-21) | 12  (6-21) | 12  (6-21) | 11  (6-20) | 11  (5-22) | 11  (5-21) | 11  (5-21) | 11  (5-21) | 9  (4–16) | 9  (5–17) | 9  (5–17) | 9  (5–17) | 7  (4- 14) | 7  (3- 13) | 7  (3- 11) | 6  (3- 11) |
| **Healthcare claim of stay, median (IQR)** | | | | | | | | | | | | | | | | |
| Cirrhosis | 2980 (2372-5886) | 3079  (2390-6077) | 3017  (2360-6215) | 2604  (2052-4934) | 2715 (640-5308) | 2764 (646-5430) | 2764 (661-5506) | 2752 (645-5565) |  |  |  |  |  |  |  |  |
| EHOF | 6262 (2970–10907) | 6497 (3073– 12017) | 6710 (3013– 12043) | 4934 (2353– 9106) | 7818 (4842-14707) | 7769 (5013-14204) | 7805 (4986-14139) | 7575 (4930 -13557) | 3760 (3760– 4013) | 3760 (3760– 4085) | 3760 (2356– 4013) | 3802 (3760– 4260) |  |  |  |  |
| **In-hospital mortality (%)** | | | | | | | | | | | | | | | | |
| Cirrhosis | 9.47 | 9.53 | 9.34 | 9.87 | 5.9 | 6 | 6.2 | 6.7 |  |  |  |  |  |  |  |  |
| Alcohol related cirrhosis | 10.11 | 10.18 | 9.89 | 10.45 | 6.7 | 6.8 | 7 | 7.4 |  |  |  |  |  |  |  |  |
| EHOF | 27.98 | 27.92 | 27.4 | 27.78 | 30.9 | 31 | 31 | 32 | 30.3 | 30.8 | 31.0 | 35.6 | 27.4 | 33.5 | 25.7 | 30.2 |
| Alcohol related EHOF | 27.99 | 28.3 | 27.45 | 28.09 | 32.6 | 32.7 | 32.4 | 33.6 | 31.0 | 32.7 | 32.2 | 37.1 |  |  |  |  |

Abbreviation: EHOF, extrahepatic organ failure

**Supplementary Table 3**. Number of hospital admissions of hepatitis C or hepatitis B patients diagnosed with cirrhosis and extrahepatic organ failure, and related proportion in all etiologies of extrahepatic organ failures

| Etiology of cirrhosis and EHOF | 2005 | 2006 | 2007 | 2008 | 2009 | 2010 | 2011 | 2012 | 2013 | 2014 | 2015 | 2016 | 2017 | 2018 | 2019 | 2020 |
| --- | --- | --- | --- | --- | --- | --- | --- | --- | --- | --- | --- | --- | --- | --- | --- | --- |
| HCV, n | 1877 | 1913 | 1823 | 1793 | 1845 | 1882 | 1748 | 1702 | 1679 | 1711 | 1629 | 1482 | 1340 | 1170 | 1410 | 1129 |
| HCV, % | 6.5% | 6.4% | 6.1% | 5.7% | 5.5% | 5.2% | 4.8% | 4.5% | 4.3% | 4.0% | 3.5% | 3.1% | 2.7% | 2.4% | 2.8% | 2.3% |
| HBV, n | 647 | 577 | 629 | 575 | 574 | 569 | 549 | 491 | 509 | 493 | 507 | 593 | 548 | 568 | 588 | 574 |
| HBV, % | 2.2% | 1.9% | 2.1% | 1.8% | 1.7% | 1.6% | 1.5% | 1.3% | 1.3% | 1.2% | 1.1% | 1.2% | 1.1% | 1.1% | 1.2% | 1.1% |
